# A Mobile App to Assist the Mentors of African American Young Men Who Have Sex With Men: Usability Study

**DOI:** 10.2196/48515

**Published:** 2023-10-27

**Authors:** Michelle R Kaufman, Kate Wright, Evan L Eschliman, Deborah Levine, Jeannette Simon

**Affiliations:** 1 Department of Health, Behavior and Society Bloomberg School of Public Health Johns Hopkins University Baltimore, MD United States; 2 Ananizach Baltimore, MD United States

**Keywords:** mentoring, youth, mobile app, men who have sex with men, MSM, HIV prevention, healthy relationships, African American

## Abstract

**Background:**

Mentoring can promote positive youth development. Owing to social and structural factors, young people in underresourced communities often lack adequate access to mentors, and naturally occurring mentors are more common than formal, programmatic mentors. There is little information on the impact of naturally occurring mentors on youth in general and even less on the role that mentors may play in promoting healthy outcomes in sexual and gender minority youth. African American young men who have sex with men (YMSM) are more likely to reside in communities with limited access to formalized mentorship programs and may benefit from naturally occurring mentoring relationships that address health outcomes, specifically related to HIV.

**Objective:**

This study is a usability test of a mobile app designed for the mentors of African American YMSM to increase mentors’ knowledge of and confidence in talking about HIV prevention and related topics with mentees.

**Methods:**

Following consent, eligible and naturally occurring mentoring pairs involving African American YMSM in Baltimore; Philadelphia; and Washington, District of Columbia, tested the app, *UrbanMentorHub,* for usability. Participants downloaded the app and used it for 1 month, completed pre- and postintervention surveys, and participated in a follow-up focus group discussion. Participants’ sociodemographic characteristics and HIV- and mentorship-related measures were characterized using descriptive statistics. Wilcoxon signed rank tests were used to test for pre- and postintervention differences in knowledge, confidence, and outcome expectancy measures. Focus group discussions were audio recorded and transcribed. Transcripts were thematically coded and analyzed to identify ways that *UrbanMentorHub* could be improved in the mentoring context.

**Results:**

Nine mentorship pairs participated in this usability study (N=18). Mentors obtained high scores on knowledge, confidence, outcome expectancies, skills, and intentions related to HIV and mentoring. No pre- or postintervention changes were observed in these measures. Mentors reported usually initiating conversations around HIV testing and pre-exposure prophylaxis; mentees and mentors equally initiated conversations on sexual practices and same-sex relationships. Mentors reported sexual practices as the most frequently discussed topic in the past month and pre-exposure prophylaxis being the least discussed. Mentees reported high comfort with HIV-related conversations. No pre- or postintervention change was observed regarding HIV knowledge. Most mentees reported having discussed most HIV-related topics with their mentor in the past month. Mentor feedback on the app was mostly neutral, although they reported positive perceptions of the idea of the app, indicating the potential for addressing a need in their communities. Mentors suggested ways to improve the app content and design elements.

**Conclusions:**

Although there was no observed statistical change in measured outcomes, and qualitative feedback was overall neutral, the results of this usability study can inform future work to design and promote interventions and resources that support mentoring relationships for African American YMSM.

## Introduction

### Mentorship and Inequities in Access to Formal and Natural Mentorship

Mentorship is defined as a relationship in which a more experienced, supportive individual is informally or formally paired with someone who offers guidance, support, and encouragement to cultivate a less-experienced individual’s positive and healthy development [1]. Mentorship can be a critical asset for a community for positive youth development [2]. Young people in low-income or underresourced communities and neighborhoods may not have the same opportunities for mentorship as their higher-income, more-resourceful peers [3,4]. A variety of social and structural forces, including racism and poverty, can result in these youth being cut off from robust community networks and resources [5-8].

If these youth do have mentors, they are more likely to be an informal or naturally occurring mentor, defined as a nonparental adult a young person identifies on their own accord and believes that they care about them and who gives them guidance, has a significant impact on them, and will be there for them when needed [9]. Studies with specific youth populations, such as those in the foster care system, have shown that natural mentorships form gradually and are less focused on achieving specific goals because the mentor is familiar to the youth and is more likely to remain in the youth’s social network [10,11]. Informal or naturally occurring mentoring relationships are one way in which sufficiently supportive social networks can be established for any youth, especially for youth with limited access to formalized mentorship programs, and may possibly improve the health and well-being of young people [12,13].

This usability study focused specifically on African American young men who have sex with men (YMSM). African American YMSM who reside in low-income, underresourced communities may have limited access to formalized mentorship programs because of a lack of existing programs with specific support for this population, including a history of exclusion from programs because of their gender or sexual identities [14]. Therefore, such youth may not benefit from the potential positive effects of mentorship on health and well-being [4,5]. In addition, men who have sex with men (MSM) experience higher rates of HIV infection, particularly YMSM who identify as Black. The higher risk for HIV alongside the lack of availability of mentoring programs encouraged this narrower focus on African American YMSM for this study [14-16].

### Mentoring of Sexual and Gender Minority Youth

A 2014 report from the National Mentoring Partnership showed that over 1.3 million, or nearly 89%, of at-risk lesbian, gay, bisexual, transgender, and queer (LGBTQ) youth (defined in the report as out of school or out of work or exhibited risk factors including regular absenteeism, poor academic performance, behavior problems in school, delinquency, and homelessness) have never had a formal mentor [17]. The study also found that at-risk LGBTQ youth were likely to want a mentor. Some formal mentoring programs are starting to address this need, as they strive to make their programs inclusive of LGBTQ youth. Big Brothers Big Sisters is committed to creating a safe environment for LGBTQ youth [18,19] and recently piloted programs specifically for sexual and gender minority (SGM) youth in several of their chapters [20].

Despite the growing interest in the mentoring of LGBTQ youth populations, few studies have measured its impact. Existing research has focused almost exclusively on formal mentoring relationships, but naturally occurring mentors may be equally important for this population [12,21]. The house-ballroom community is a unique social network that often functions as a surrogate family for SGM youth, particularly Black gay and bisexual men as well as Black transgender, nonbinary, and gender nonconforming youth. House mothers or fathers serve as natural mentors for SGM youth who are exploring sexual behaviors and gender presentation [22]. However, the extent to which these social networks foster protective and health-seeking behaviors among the youth remains unclear [23]. In addition, the lack of research on the associations between naturally occurring mentorship pairs and HIV prevention [24] as well as the limited access to formal mentorship for this youth population warrants attention in HIV prevention research efforts.

### Mentoring to Promote Health

Previously proposed models have attempted to explicate the relationships between mentorship and health outcomes [25-27], but overall, the science of mentoring as prevention and its effects on adolescent and young adult health outcomes (particularly HIV-related outcomes) is limited [28-30]. Specifically, the health-related relationships in these models are rarely empirically tested, leaving a gap in the literature regarding the specific processes by which mentoring’s interpersonal nature can serve as an effective intervention and health promotion tool. Often, evaluations of well-regarded, successful mentorship programs do not focus on health-related outcomes [31]. However, some findings of controlled evaluations of the Big Brothers Big Sisters mentoring programs, including a large-scale randomized controlled trial [32], allow for cautious optimism about the potential viability of mentoring interventions to improve a variety of health outcomes, including illegal drug use initiation [33], injury prevention (ie, conflict avoidance self-efficacy and not engaging in fights) [34], and mental health (ie, depression and anxiety symptoms) [32]. It is plausible to frame some of these outcomes as upstream HIV prevention factors [35], yet few studies explicitly link mentoring to HIV-related outcomes [36-38], let alone HIV outcomes in SGM youth and African American YMSM in particular.

### Study Rationale and Objectives

Previous research and evaluation show that youth mentoring relationships have the potential to positively affect youth behaviors. For a hard-to-reach population, such as African American YMSM, trusted adults who provide socioemotional support, encouragement, and motivation and who follow-up with youth on a regular basis to hold them accountable for healthy behaviors may be productive in improving HIV-related outcomes. However, mentors need tools (eg, apps and other technology [39]) to effectively mentor on HIV prevention and related issues, such as family rejection, mental health, substance use, healthy relationships, and financial stability [14].

This paper describes the usability testing of the mobile app *UrbanMentorHub* to assess its ability to increase mentors’ knowledge of and confidence in talking about HIV prevention and related sensitive issues with mentees. Given the high rates of HIV in Black MSM communities [15,16] and that HIV was the focus of the funding mechanism supporting this work, the app’s content was primarily relevant to HIV prevention and treatment. Other related content to promote the health and well-being of Black YMSM was added based on formative research [14]. This app is designed to assist the mentors of African American YMSM in addressing HIV and related issues as part of their mentoring relationship. Our hypothesis was that if mentors were given a digital tool to educate themselves on HIV, build their mentoring skills and efficacy, and provide them with content to catalyze mentoring conversations around HIV-related topics, their mentoring relationships would have a positive impact on African American YMSM and their health.

## Methods

### Conceptual Framework

The development of *UrbanMentorHub* was based on social cognitive theory, which is the cognitive formulation of the social learning theory by Bandura and Walters [40]. It explains human behavior through a 3-way, reciprocal, and dynamic model in which personal factors, environmental influences, and behavior constantly interact. This theory posits that people learn not only from their own experiences but also by observing others’ actions and the results of those actions. The key components of this theory are outcome expectancy and self-efficacy, both of which were central to the development of *UrbanMentorHub*. Outcome expectancy refers to the assignment of a value to the outcomes of behavior change. Self-efficacy refers to a person’s confidence in their ability to act and persevere in that action regardless of the challenges or obstacles they might encounter. Self-efficacy influences expected outcomes of behavior, and expected outcomes also influence self-efficacy judgments [41-43]. *UrbanMentorHub* aims to improve self-efficacy and positive outcome expectancies around HIV-related outcomes via the mentor and mentee relationship, and it models mentoring interactions when a mentor addresses HIV-related issues.

### User-Centered Design Research

A user-centered design approach was critical for the app development process. This allowed us to meet the needs of, increase interest from, and improve usability for both the mentor and mentee end users [44-46]. First, we conducted in-depth interviews with African American SGM youth mentees (n=17) and mentors to African American SGM youth (n=20) in 3 mid-Atlantic cities (Baltimore; Philadelphia; and Washington, District of Columbia). These interviews explored how and to what extent topics related to sexual health and HIV were broached and discussed, mentors’ knowledge and confidence in mentoring on these topics, and the hypothetical acceptability and appropriateness of a mobile app in facilitating these discussions. The interview findings have been described in detail elsewhere [14]. Major findings showed that these discussions, once initiated, are perceived positively; that mentors want to be more knowledgeable about sexual health and HIV; and that an app could be useful, particularly one that takes mentees’ multifaceted needs into account and provides mentors with self-training, mentorship tips, sexual health and HIV resources, and support from other mentors [14].

Subsequently, mock-ups of the app screens and sample content were reviewed by a focus group made up of mentoring program staff, mentors, and key stakeholders working with African American YMSM in each city. The focus group participants were asked to review the content and provide feedback on whether it was applicable to the mentoring of African American YMSM, features and content that would be most compelling, and what might be missing to further encourage HIV-related mentoring discussions. The combined results of the in-depth interviews and focus group reactions to mock-ups were used to build the initial app framework and content.

### Description of the App

*UrbanMentorHub* is an early phase web app designed to assist mentors in urban areas by introducing and maintaining mentoring with youth around sensitive issues. The early phase version was built using out-of-the-box software without a professional graphic designer to reduce costs in the development phase. Version 1 of *UrbanMentorHub* is designed to assist mentors of YMSM living in urban areas of the United States. This study focused on usability testing of the app to improve mentoring, primarily on HIV-related outcomes. However, the app contains four content-focused areas related to HIV risk: (1) HIV and pre-exposure prophylaxis (PrEP); (2) mental health; (3) financial insecurity, including the subtopics of substance use and transactional sex; and (4) rejection from families. These topics were identified during the user-centered design phase as areas related to potential HIV risk behavior among this population of young men.

**Figure 1:**
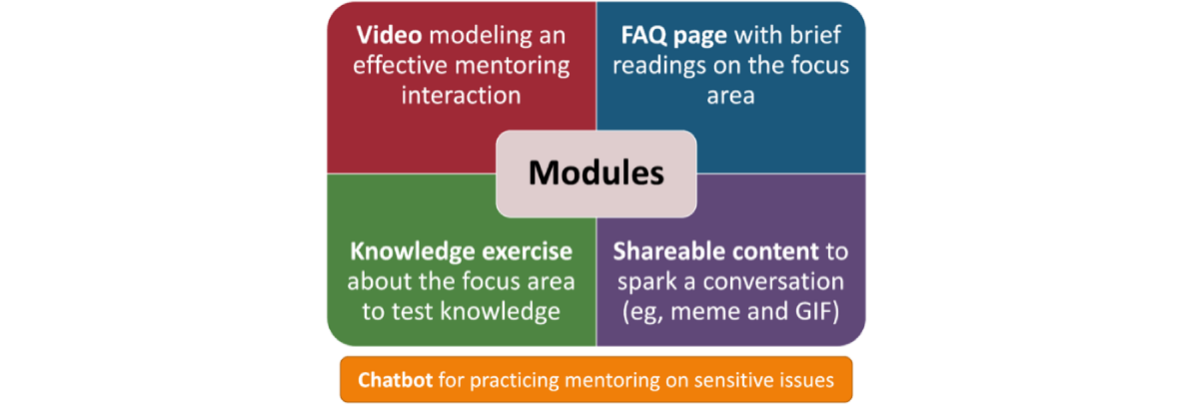
UrbanMentorHub features. FAQ: frequently asked questions.

The app’s modules introduce mentors to these topics and provide resources to improve their mentoring regarding the selected issues. Each module contains the following: (1) a video modeling an effective mentoring interaction around the topic, where a mentor and mentee discuss an issue and model best practices for active listening, mentoring without judgment, and supportive language; (2) a frequently asked questions page with brief readings to help the mentor understand the topic in detail; (3) a knowledge exercise about the focus area so that mentors can test their own knowledge of the issue; and (4) a piece of sharable content that mentors can share via social media or directly with their mentee to help spark a conversation on the topic (Figure 1). The sharable content could include a meme, GIF, short video, or photo. Additional features within the app include a forum where mentors can share ideas with each other and a mentoring chatbot with which mentors can practice mentoring on sensitive issues. The chatbot mimics what a conversation on these sensitive topics may look like when talking to a young person.

The primary purpose of the app is to make it easier for mentors to learn about these sensitive topics so that they have the information they need when having conversations that could affect the long-term health of their mentees. The module approach allows for the content to be available as a library; therefore, mentors can easily access content and learn about a particular topic if the need arises in their mentoring relationship.

### Recruitment

Mentoring pairs in Baltimore; Philadelphia; and Washington, District of Columbia, participated in this study. Following consent, eligible and naturally occurring mentoring pairs involving African American YMSM of varying ages or developmental levels were asked to access the web app and use it for a 1-month period. These pairs were also asked to complete pre- and postintervention surveys and to participate in a follow-up focus group to discuss their impressions of the app.

Mentors eligible to participate in the usability test were nonfamilial adults mentoring cisgender YMSM in a naturally occurring setting (ie, not affiliated with a formal mentoring program). The mentors were in a mentoring relationship with an eligible mentee for at least 3 months before the usability testing (to allow time for sufficient relationship building to occur at the beginning of a mentoring match) and had a mentee who agreed to participate in the usability study. The sexual and gender identity of the mentor were not eligibility factors.

Mentees eligible to participate in the usability study were urban African American cisgender male individuals who were aged 15 to 24 years; had sexual interest in males or reported currently having sex with men during the screening; were in a naturally occurring mentoring relationship and passed a mentoring relationship screener (to determine whether the relationship was indeed a mentoring relationship); were in a mentoring relationship for at least 3 months before the usability test (to allow time for trust and rapport to build with their respective mentors); and had a mentor who agreed to participate.

We worked with historically Black colleges and universities, LGBTQ youth–focused organizations, and house-ballroom community members in each city to recruit participants. Recruitment flyers and advertisements were also distributed at relevant community events strategically placed at relevant university locations; posted on social media sites; and advertised on radio shows, in newspapers, and on gay dating apps. Additional participants were recruited through respondent-driven sampling. Participants received US $10 for each pair they recruited (up to 5 pairs and US $50) who completed the study process.

When a mentee contacted us with interest in the study, we asked them to provide contact information for their mentor or have the mentor contact us directly and vice versa if a mentor made initial contact with the study team. One party’s participation was contingent on the other party’s participation. Both mentors and mentees who expressed interest in the study were screened for eligibility.

### Usability Study Implementation Procedure

The usability study took place over a span of approximately 1 month for each participant. All participants were asked to meet in a group setting—either in person or remotely due to the COVID-19 pandemic—both before and after the study period for a short training, pre- and postintervention surveys, and a focus group.

#### Training, Overall Use Period, and Reminder Messages

Mentors downloaded the app and received a short training (30 min) on how to use its features. They used the app for 1 month, during which time they received a maximum of 8 SMS text messages from the study team to remind them of available content. Messages were pushed through the project phone number via automated texting to the participant’s cell number on file. An example of text content was “UrbanMentorHub here! Did you know that daily PrEP reduces the risk of getting HIV from sex by more than 90%? To learn more about PrEP, sexual health, and how this might affect your mentee, click here.”

#### Pre- and Postintervention Surveys

Before and after the usability test period, participants took surveys that assessed whether the mentoring pair discussed issues relevant to HIV and, in the case of the mentor, how confident and knowledgeable they felt in mentoring on these topics. The postintervention survey also assessed their personal reactions to the content, including comfort level, relevance to their own life or mentoring relationship, and whether the content was helpful. Mentee surveys asked whether the mentor discussed any of the issues with the mentee or shared with him any of the social media content (GIFs or memes) available in the app. These measures are described in detail in subsequent sections.

#### Focus Groups

Immediately after completing the postintervention survey, the mentors and mentees were brought together to participate in a focus group regarding the general usability and feasibility of the app to enhance mentoring. Mentors were asked to provide feedback on any content that was missing or should be removed, how easy or difficult the app was to use, whether they liked the content format, and how to improve the app experience in the mentoring context. Mentees were asked whether the mentor talked with them about the app content or shared any of the social media content, how the conversations went, and what they thought the mentor could change to improve such conversations in the future. All focus groups were audio recorded.

### Ethical Considerations

All study procedures were approved by the Institutional Review Board at the Johns Hopkins Bloomberg School of Public Health (approval number 00008197). Each study participant read and signed an informed consent form outlining the purpose, risks, and benefits of the study; participants had the opportunity to ask questions and seek clarification from the study staff when reading and signing the form. The informed consent form also described how participation and the resulting data would be kept private and confidential (ie, all study data were deidentified and stored in either locked drawers or on password-protected servers accessible only to the study team). Mentors and mentees who participated in the usability feedback session each received a US $50 gift card. Any mentor-mentee pair with both participants attending the usability feedback session was entered into a raffle to win 2 event tickets (eg, sporting event, play, or concert) worth US $200 with the intention that they attend the event together. Each participant could also receive US $10 for recruiting additional mentoring pairs (up to 5 pairs and US $50) who completed the study process.

### Measures

#### Sociodemographics

Both mentors and mentees were asked about their age, education level, city of residence (ie, Baltimore; Philadelphia; or Washington, District of Columbia), gender, and sexual identity. Employment and occupation were assessed using an open-ended question for mentors and as a set of multiple-choice options for mentees. Owing to results from our formative research [14], transgender and nonbinary individuals were not included in this usability study; therefore, anyone who identified as transgender or nonbinary would not continue with the process beyond this point.

#### Mentorship Length

Both mentors and mentees reported the duration of their mentoring relationship; mentors were only asked how long they have been a mentor to any young person.

#### HIV Knowledge

A 3-item HIV knowledge scale was used to assess both mentors’ and mentees’ knowledge about how to prevent HIV infection, PrEP, and HIV testing [47]. Responses were coded as either correct or incorrect and summed for a total knowledge score of 0 to 3.

#### Mentoring Self-Efficacy

Mentors were asked a series of 12 questions related to their self-efficacy, confidence, and comfort when talking to their mentee in general and regarding specific HIV-related topics (ie, risk behaviors, transmission of HIV, HIV testing, PrEP, safer sex, and same-sex relationships). Responses were given on a scale of 1 (ie, I completely disagree or not at all comfortable) to 10 (ie, I completely agree or completely comfortable). The scores ranged from 12 to 120, and the internal consistency of this scale was strong (pretest Cronbach α=.86; posttest Cronbach α=.95).

#### Mentoring Outcome Expectancy

Mentors’ outcome expectancy related to talking to their mentee about sex and sexual health was measured using 10 items adapted from a scale originally used for parents of older children and adolescents [48]. Items were presented under the heading “If you talk with your mentee about sex and sexual health, how sure are you of the following?” Participants were asked to select a response on a 5-point Likert scale from “not at all sure” to “completely sure.” Some items included “you will feel like a responsible mentor,” “your mentee will be less likely to engage in risky behavior,” and “it will make your relationship with your mentee stronger.” Scores for this scale ranged from 10 to 50, and internal consistency was fair in this sample (pretest Cronbach α=.76; posttest Cronbach α=.78).

#### HIV-Related Mentoring Outcome Expectancy

Mentors’ outcome expectancy specific to HIV-related mentoring was measured using a construction similar to the general mentoring outcome expectancy scale. The HIV-related scale was made up of 4 items asking how likely initiating a conversation about HIV will be well received by the mentee and lead to the mentee getting tested for HIV, as well as how likely initiating a conversation about PrEP will be well received by the mentee and lead to the mentee talking to a health care professional. Items were rated on a 10-point Likert scale ranging from “not at all” to “certainly.” This scale ranged from 4 to 40 and had a high internal consistency (pretest Cronbach α=.90; posttest Cronbach α=.90).

#### Mentoring Skills

Mentoring skills were assessed using a 10-item scale adapted from the 8-item Mentoring Competency Assessment [49]. Mentors rated their skills in domains such as “active listening” and “helping the mentee develop strategies to meet goals” on a 10-point Likert scale ranging from “not skilled at all” to “extremely skilled.” Two additional skill domains (ie, “talking about sexual health” and “talking about healthy relationships”) were added for the purposes of this study. Scores ranged from 10 to 100, and the internal consistency of this scale was relatively low for the pretest (Cronbach α=.63) but extremely high for the posttest (Cronbach α=.99).

#### Mentoring Intentions

The mentors’ intentions to have conversations about HIV testing, PrEP, sexual practices, and same-sex relationships were measured using 4 items with the stem of “How likely are you to have a conversation with your mentee about [topic] in the next month?” Response options were on a 10-point Likert scale from “not at all likely” to “definitely.” These items were analyzed separately by topic.

#### Mentoring Practice

For mentors, 4 sets of 2 items each were asked about conversations the mentor and mentee had about HIV testing, PrEP, healthy sexual practices, and relationships with other men. For each of the topics, mentors were asked who usually introduced the conversations and how often the topic was discussed in the past month. Mentees were asked how comfortable they were talking with their mentor about each of the 4 topics (10-point Likert scale from “not at all comfortable” to “completely comfortable”) and the frequency of discussions in the past month. In addition, mentees were asked about the frequency of discussions in the past month on mental health concerns (eg, feeling sad, depressed, anxious, or suicidal); how to handle stress; and violence in the mentees’ family or neighborhood. These items are reported separately by topic.

#### Mentor App Feedback

In the postintervention survey, mentors were asked their rating of various features and areas of content in the app and responded on a 4-point scale of “I don’t like it,” “It’s OK,” “I like it,” and “It’s amazing!” They were also asked which feature they preferred. Mentors were then asked a variety of questions related to the acceptability of the app, including how much they disagree or agree (ie, using a 10-point Likert scale) with a variety of statements on app usability and usefulness of features, whether the app provided the mentor with new knowledge, whether it made them more confident or comfortable in their mentoring relationship, and if they would share or recommend the app.

#### Mentor Feedback on Mentoring Relationship

Mentors were asked if there were other topics on which they wish to improve their mentoring. They were also given space to provide any feedback on discussing sensitive topics with mentees they wished to share.

#### Mentee App Feedback

In the postintervention survey, mentees were asked what, if any, app content they were shown. For any content they were shown, mentees were asked how they would rate it on a 4-point scale of “I don’t like it,” “It’s OK,” “I like it,” and “It’s amazing!” and which content was their favorite. Mentees were then shown a screenshot of a breathing exercise and a GIF, both from the app, and asked if they had seen them. If yes, they were asked to rate the items on the same 4-point scale.

#### Mentee Feedback on the Mentoring Relationship

Mentees were asked if there are other topics they wanted their mentor to discuss with them, as well as any overall feedback about their mentoring relationship they wished to share.

### Analysis

Descriptive statistics were calculated to assess the participants’ sociodemographic characteristics, HIV prevention knowledge and behaviors, and mentorship-related constructs such as self-efficacy and mentorship experiences. Wilcoxon signed rank tests (ie, for nonparametric pairwise comparisons) were used to test for statistically significant differences in mentors’ HIV knowledge, confidence in mentoring (self-efficacy), general mentoring outcome expectancy, HIV-related mentoring outcome expectancy, and mentoring skills before and after as well as mentees’ HIV-related knowledge before and after app use. The results were considered significant if *P*<.05. Quantitative analyses were performed using Stata 17 (StataCorp) [50].

For qualitative analyses, the focus groups were audio recorded and transcribed, and the text from each group was thematically coded. The choice to use a single coder who was not previously involved in the research (ELE) was informed by pragmatic considerations, including personnel availability, the relatively small amount of data, and the desired outcomes of the analysis (ie, to understand perceptions of the app and ways participants thought *UrbanMentorHub* can be improved) [51].

## Results

### Participant Characteristics

We recruited 4 mentorship pairs (8 participants) in Baltimore and 5 mentorship pairs (10 participants) in Philadelphia, resulting in a total of 18 participants. Recruitment from Washington, District of Columbia, was not feasible for this usability study. This was likely related to interruptions in relationship building with the community and recruitment efforts because of the COVID-19 pandemic. Of the 18 recruited participants, 4 mentoring pairs from Baltimore and 4 pairs from Philadelphia completed at least one survey, resulting in a total of 16 participants who completed either the preintervention, postintervention, or both surveys (see Table 1 for demographic characteristics).

**Table 1:** Sociodemographic characteristics of mentors and mentees.

Characteristics			Mentors (n=8)		Mentees (n=8)
Age (y; n=7), mean (SD)			34.3 (9.8)		23.3 (1.1)
**Education, n (%)**					
	High school	0 (0)		4 (50)	
	Some college, no degree	2 (25)		2 (24)	
	Associate’s degree	1 (13)		1 (13)	
	Bachelor’s degree	3 (38)		0 (0)	
	Master’s degree	2 (25)		0 (0)	
	Professional degree	0 (0)		0 (0)	
	Doctoral degree	0 (0)		0 (0)	
	Missing	0 (0)		1 (13)	
**Employment, n (%)**					
	Unemployed	0 (0)		3 (38)	
	Student	0 (0)		1 (13)	
	Self-employed	2 (25)		0 (0)	
	Employed part time	0 (0)		1 (13)	
	Employed full time	6 (75)		2 (25)	
	Missing	0 (0)		1 (13)	
**Current city, n (%)**					
	Baltimore	4 (50)		4 (50)	
	Philadelphia	4 (50)		3 (38)	
	Washington, District of Columbia	0 (0)		0 (0)	
	Missing	0 (0)		1 (13)	
**Gender, n (%)**					
	Female	1 (13)		0 (0)	
	Male	7 (88)		7 (88)	
	Missing	0 (0)		1 (13)	
**Sexual orientation, n (%)**					
	Heterosexual	1 (13)		0 (0)	
	Homosexual	6 (75)		5 (63)	
	Bisexual	1 (13)		2 (25)	
	Missing	0		1 (13)	
Length of time mentoring in general in years, mean (SD)			11.9 (7.4)		N/A^a^
Length of time mentoring mentee in study (n=6), mean (SD)			1.4 (1.6)		2.4 (3.9)

^a^N/A: not applicable.

### Quantitative Pre- and Postintervention Results

#### Mentors

Mentors reported high levels of HIV knowledge, high confidence in mentoring (self-efficacy), high general and HIV-related mentoring outcome expectancies, high mentoring skills, and high mentoring intentions for each HIV-related topic (ie, HIV testing, PrEP, sexual practices, and same-sex relationships). No statistically significant pre- or postintervention differences were observed in mentors’ HIV knowledge (*P*=.99), self-efficacy (*P*=.22), mentoring outcome expectancy (*P*=.22), HIV-related mentoring outcome expectancy (*P*=.59), or mentoring skills (*P*=.35). In general, in both pre- and postapp use, mentors reported that they usually initiated conversations around HIV testing and PrEP, whereas conversations on sexual practices and same-sex relationships tended to be initiated about equally by the mentor and mentee. Mentors reported sexual practices as the most frequently discussed topic in the past month and PrEP being the least discussed (Table 2).

**Table 2:** Pre- and postapp use outcomes for mentors.

Construct (range of possible scores)			Before the app use (n=8)	After the app use (n=5)
HIV knowledge (0-3), mean (SD)			2.9 (0.4)	3.0 (0.0)
Confidence in mentoring (self-efficacy; 12-120), mean (SD)			104.0 (14.3)	94.2 (25.2)
Mentoring outcome expectancy (10-50), mean (SD)			45.0 (3.6)	38.6 (4.5)
HIV-related mentoring outcome expectancy (4-40), mean (SD)			34.1 (6.1)	32.4 (7.6)
Mentoring skills (10-100), mean (SD)			92.5 (4.6)	81.2 (20.5)
**Mentoring intentions, mean (SD)**				
	HIV testing (1-10)		8.3 (2.0)	7.8 (1.9)
	PrEP^a^ (1-10)		8.1 (2.1)	7.8 (1.9)
	Sexual practices (1-10)		8.5 (1.7)	8.0 (2.1)
Same-sex relationships (1-10), mean (SD)			8.6 (1.4)	8.4 (2.1)
**Mentoring practice, who initiates conversations, n (%)**				
	**HIV testing**			
		Usually me (mentor)	4 (50)	3 (60)
		Usually my mentee	0 (0)	0 (0)
		We both do it equally	3 (38)	2 (40)
		We do not talk about this topic	1 (13)	0 (0)
	**PrEP**			
		Usually me (mentor)	5 (63)	3 (60)
		Usually my mentee	0 (0)	0 (0)
		We both do it equally	2 (25)	2 (40)
		We do not talk about this topic	1 (13)	0 (0)
	**Sexual practices**			
		Usually me (mentor)	2 (25)	2 (40)
		Usually my mentee	0 (0)	0 (0)
		We both do it equally	6 (75)	3 (60)
		We do not talk about this topic	0 (0)	0 (0)
	**Same-sex relationships**			
		Usually me (mentor)	2 (25)	1 (20)
		Usually my mentee	1 (13)	0 (0)
		We both do it equally	5 (63)	4 (80)
		We do not talk about this topic	0 (0)	0 (0)
**Mentoring practice, times discussed in past month, n (%)**				
	**HIV testing**			
		Never	2 (25)	1 (20)
		1-2 times	4 (50)	4 (80)
		3-5 times	1 (12)	0 (0)
		≥6 times	1 (12)	0 (0)
	**PrEP**			
		Never	3 (38)	1 (20)
		1-2 times	4 (50)	3 (60)
		3-5 times	1 (13)	1 (20)
		≥6 times	0 (0)	0 (0)
	**Sexual practices**			
		Never	0 (0)	0 (0)
		1-2 times	3 (38)	2 (40)
		3-5 times	2 (25)	2 (40)
		≥6 times	3 (38)	1 (20)
	**Same-sex relationships**			
		Never	0 (0)	0 (0)
		1-2 times	4 (50)	3 (60)
		3-5 times	2 (25)	1 (20)
		≥6 times	2 (25)	1 (20)

^a^PrEP: pre-exposure prophylaxis.

#### Mentees

Mentees reported high HIV knowledge as well as high comfort in conversations with their mentors around HIV testing, PrEP, sexual practices, and same-sex relationships. No statistically significant difference was observed between HIV knowledge pre- and postapp use (*P*=.16). Both before and after the intervention, most mentees reported having discussed 5 of the 7 topics asked (ie, HIV testing, PrEP, sexual practices, same-sex relationships, and how to handle stress) with their mentor in the past month. Mental health concerns were discussed at least once in the past month by a majority of participants before the intervention but not after the intervention, and a majority of mentees at both time points reported having never talked to their mentor about family or neighborhood violence in the past month (Table 3).

**Table 3:** Pre- and postapp use outcomes for mentees.

Construct (range of possible scores)				After the test (n=8)		Before the test (n=4)
HIV knowledge (0-3), mean (SD)				2.3 (1.0)		3.0 (0.0)
**Mentoring practice, comfort in conversations^a^ (1-10), mean (SD)**						
	HIV testing			9.3 (1.9)		10.0 (0.0)
	PrEP^b^			9.9 (0.4)		10.0 (0.0)
	Sexual practices			8.7 (2.2)		10.0 (0.0)
Same-sex relationships, mean (SD)				9.4 (1.1)		9.3 (1.5)
**Mentoring practice, times discussed in past month^a^, n (%)**						
	**HIV testing**					
		Never	1 (14)		2 (50)	
		1-2 times	3 (43)		0 (0)	
		3-5 times	3 (43)		2 (50)	
		≥6 times	0 (0)		0 (0)	
	**PrEP**					
		Never	2 (29)		1 (25)	
		1-2 times	2 (29)		2 (50)	
		3-5 times	2 (29)		1 (25)	
		≥6 times	1 (14)		0 (0)	
	**Sexual practices**					
		Never	2 (29)		0 (0)	
		1-2 times	1 (14)		2 (50)	
		3-5 times	3 (43)		2 (50)	
		≥6 times	1 (14)		0 (0)	
	**Same-sex relationships**					
		Never	1 (14)		0 (0)	
		1-2 times	1 (14)		3 (75)	
		3-5 times	2 (29)		1 (25)	
		≥6 times	3 (43)		0 (0)	
	**Mental health concerns**					
		Never	2 (29)		3 (75)	
		1-2 times	2 (29)		1 (25)	
		3-5 times	1 (14)		0 (0)	
		≥6 times	2 (29)		0 (0)	
	**How to handle stress**					
		Never	2 (29)		1 (25)	
		1-2 times	2 (29)		2 (50)	
		3-5 times	1 (14)		1 (25)	
		≥6 times	2 (29)		0 (0)	
	**Family or neighborhood violence**					
		Never	4 (57)		3 (75)	
		1-2 times	1 (14)		1 (25)	
		3-5 times	1 (14)		0 (0)	
		≥6 times	1 (14)		0 (0)	

^a^Before the test, n=7.

^b^PrEP: pre-exposure prophylaxis.

### App Feedback

#### Mentors

The 5 mentors who completed the postapp use survey provided overall neutral feedback on the various features. The most common response across all 6 content domains was “It’s OK,” although 1 mentor responded with mostly “It’s amazing” and “I like it” and 1 mentor responded with mostly “I don’t like it.” The ratings of the *UrbanMentorHub* app overall were most commonly “It’s OK” (3/5, 60%), with the other 2 mentors responding, “It’s amazing” (1/5, 20%) and “I don’t like it” (1/5, 20%).

#### Mentees

Among the 4 mentees who completed the postapp use survey, none reported seeing any of the sharable app content. One mentee reported seeing and liking the frequently asked questions pages, which were not part of the intended sharable content provided to mentors.

### Mentoring Relationship Feedback

#### Mentors

Five mentors identified a range of other topics on which they wanted to improve their mentoring. These topics included job skills (n=5), mental health (n=3), family issues (n=3), transitioning to college (n=3), housing (n=3), drug use (n=3), sexual identity (n=2), gender identity (n=2), school (n=2), alcohol use (n=1), and tobacco use (n=1).

#### Mentees

Of the 3 mentees who expressed interest in additional topics they wanted to discuss with their mentors, the topics again ranged widely and included mental health (n=2), family issues (n=2), stress (n=1), sexual identity (n=1), gender identity (n=1), school (n=1), race and racism (n=1), housing (n=1), and alcohol use (n=1).

### Qualitative Results

In the 4 focus groups following the usability study period, mentors largely reported positive perceptions of the hypothetical idea of an app, such as *UrbanMentorHub*, believing it addresses a clear need in their communities. Mentors stressed the importance of mentorship for African American YMSM and noted the lack of mentorship programs specifically for this population:

What’s happening within our community is there are several mentorship programs that work with Black boys. But there are none...that really educate or work with Black boys who identify as LGBTQ. And, so, we can work with Black boys who identify as LGBTQ...They’re talking about HIV, they’re talking about AIDS, they’re talking about all of that in all these other programs, but none of the young people are being mentored by anybody...they’ll talk about this, but then they’ll leave and there’s nobody they can connect to after that.

Many mentors saw promise in an app to provide information and resources for mentors to address sensitive topics with their mentees beyond only HIV:

Maybe you might be afraid to talk about them to your mentee, but these things, they’re not as hard as they seem to be. There is a clear guidance on how to conduct your mentee through

these conversations

Regarding the *UrbanMentorHub* app itself, most mentors agreed that the app was easy to use, although some mentors felt that the design was boring and uninviting. Almost all mentors expressed at least some disappointment or dislike related to the content of the app and offered several suggestions for improvement in specific content and design elements. These included changing the name (ie, in a way that eliminates the vague and racially coded word urban), making the video vignettes of mentoring conversations more “realistic,” and expanding the content provided to include things that “make you want to use [the app]” (eg, news and current events, more relevant memes, and social networking aspects). More experienced mentors also highlighted that the current version and scope of content would be more useful to new or less-experienced mentors.

As for the mentee perspective, no mentee reported that the app was mentioned to them in a positive light; if the app was mentioned to the mentee, it was alongside a negative perception (eg, “it’s fake” or “it’s not good”). Both mentors and mentees viewed the inclusion of information on mental health and substance use favorably, explaining that these non-HIV topics are often given less attention and resources.

## Discussion

### Principal Findings

Although usability studies, including those for digital health interventions [52,53], can provide important insights for future intervention, their potential benefit is often limited by their results not being fully and transparently reported [53,54]. As such, although the quantitative findings of this study did not indicate a clear impact of app use on any of the measured outcomes, some findings are worth highlighting. The quantitative outcomes reported before and after app use indicated that both mentors and mentees in this sample possessed high levels of HIV knowledge and comfort in their mentoring relationships. Mentors also reported high levels of mentoring self-efficacy, mentoring skills, and intention to talk about all 4 probed topics of interest (ie, HIV testing, PrEP, sexual practices, and same-sex relationships). Taken together and across time points, the mentoring practice results suggest that mentors and mentees discuss sexual practices and same-sex relationships readily and frequently, but they discuss HIV testing and PrEP less, despite mentees reporting high levels of comfort in discussing these topics with their mentors. Notably, the internal consistency of the adapted and novel measures may suggest suitability of these measures in assessing the selected constructs for this sample. Despite the lack of statistical changes observed between the pre- and postapp use periods, the documentation of these outcomes adds to the nascent evidence base on mentoring for African American YMSM and SGM youth [5,39].

Regarding app feedback, quantitative feedback was mixed, particularly among mentors who had a higher rate of having viewed app content. For mentees, quantitative app feedback was especially sparse, given the low number of mentees who were shown app content by their mentors. However, quantitative feedback on mentoring relationships did provide some clear indications that mentors and mentees both expressed a range of topics of interest for conversation. Importantly, many of these topics were of interest to both parties, namely mental health, family issues, sexual identity, gender identity, and school (including transitioning to college). These topics, as well as mentor-identified topics of interest such as job skills, housing, and substance use, offer future domains of content that could be relevant to and resonate with mentors of African American YMSM. Qualitative data also supported the importance of the provided resources and information on relevant topics in addition to HIV. This participant emphasis on covering many topics mirrors the formative research for this app, concepts such as HIV prevention fatigue and other findings suggesting that overly HIV-focused interventions often neglect other important concerns of priority populations such as African American YMSM [14,55-57].

The qualitative data in this study help contextualize the quantitative results, and together, the findings point toward the promise of an app intended to support mentors of African American YMSM with health-related conversations. Although participants reported reservations and concerns about the beta version of *UrbanMentorHub*, the general idea of an app to support new and early stage, naturally occurring mentoring relationships for African American YMSM and their mentors appears to fill an expressed need. The changes recommended in the qualitative results also highlight how important a user-centered design approach is to the development and implementation of an app [45]. Although this beta version was informed by extensive formative qualitative research [14], participants still identified desired changes in many aspects (eg, the app name, the stilted scripted nature of the mentoring video vignettes, and the lack of invitingness of the user interface). Without this crucial step of beta testing, the app would have lacked sufficient relevance for its target audience or even alienated potential users through its name or design. As more programs for African American YMSM and other SGM youth mentorship are being developed and evaluated [20,21], it is critical to seek and respond to feedback from mentors and mentees across stages of program development, implementation, evaluation, and sustainment [58]. In addition, the budget for a robust, user-centered design process and graphic designer should be included in any future apps to be developed for health purposes.

### Limitations

This usability study had some limitations. First, we encountered multiple barriers with recruitment efforts. Although participants were initially recruited before the COVID-19 pandemic for in-person app training and subsequent data collection, recruitment efforts and app testing beginning in March 2020 had to be moved exclusively to web-based means. The pandemic also interrupted our ability to meet and grow collaborations with organizations that support SGM in the 3 cities. This made the recruitment of such youth participants particularly challenging, especially given the social and structural forces that can make African American SGM youth hidden [58,59]. In addition, this youth population may not have consistent phone numbers, housing, or employment, making it difficult to stay connected with participants. Organization staff supporting mentor and mentee recruitment to test the app were also subject to change without notice, causing delays and, on occasion, no response. Altogether, these difficulties resulted in a relatively small number of participants (N=18), only 16 of whom completed at least 1 survey; this small sample size limited the quantitative detection of any changes in the outcomes measured.

Second, our eligibility criteria required mentors and mentees to have had a relationship for a minimum of 3 months. Most participating mentors had been mentoring for an average of 12 years and with their paired mentee included in the study for over 1 year. Mentors stated that this app may be more useful for newer mentors, which should be explored in future research.

Finally, we did not have the funding to fully develop a mobile app with advanced graphics and features. Instead, we created a web app that can run on any type of smartphone with network or Wi-Fi access, as it was less expensive to develop because it used out-of-the-box features. As we did not include a graphic designer due to resource limitations and web apps having fewer features available, this impacted our ability to include certain design elements and may have contributed to the lack of enthusiasm expressed by participating mentors.

### Conclusions

Reporting the results of the usability study of the *UrbanMentorHub* app is a small step toward reducing inequities in mentorship programming faced by African American YMSM and SGM youth. Although the hypothesized impacts were not detected in this quantitative analysis, findings from this multimethod usability study documented outcomes related to naturally occurring mentoring relationships among this population, highlighted the perceived importance of an app for mentors of African American YMSM, and underscored the critical need for a robust user-centered design process to ensure the acceptability and usability of mentorship apps. Future work can build on these findings to create resources that leverage naturally occurring mentoring relationships, empower mentors of African American YMSM to have crucial conversations around a variety of health- and HIV-related topics, and ultimately help improve African American YMSM’s health and well-being.

Acknowledgments

The authors are immensely grateful to the participants who provided feedback on the development of this app. They also thank Drs Renata Sanders, Carl Latkin, Nick Ialongo, and David DuBois for their mentorship in this line of work. The authors would also like to thank James Conley III for assisting with early recruitment; Kim Dam and Albert Casella for their research coordination assistance early in the project; and Samantha Tsang for helping structure the manuscript.

This research was funded by an Administrative Supplement to the Johns Hopkins University Center for AIDS Research, National Institutes of Health (NIH)–funded program (P30AI094189), which is supported by BI, NIDA, NIHMD, NIA, NIGMS, and NIDDK. This research was also supported by the National Institute on Drug Abuse under award K01DA042138. The content is the sole responsibility of the authors and does not necessarily represent the official views of the NIH.

## Abbreviations

LGBTQ: lesbian, gay, bisexual, transgender, and queer
MSM: men who have sex with men
PrEP: pre-exposure prophylaxis
SGM: sexual and gender minority
YMSM: young men who have sex with men

## Data Availability

The data generated or analyzed during this study are available from the corresponding author upon reasonable request.
